# Psychiatric disorder in early adulthood and risk of premature mortality in the 1946 British Birth Cohort

**DOI:** 10.1186/1471-244X-11-37

**Published:** 2011-03-08

**Authors:** Max Henderson, Matthew Hotopf, Imran Shah, Richard D Hayes, Diana Kuh

**Affiliations:** 1 Clinical Senior Lecturer in Epidemiological and Occupational Psychiatry King's College London, Institute of Psychiatry Department of Psychological Medicine Weston Education Centre Cutcombe Road London SE5 9RJ UK; 2 King's College London, Institute of Psychiatry Department of Psychological Medicine Weston Education Centre Cutcombe Road London SE5 9RJ UK; 3 MRC National Survey of Health and Development MRC Unit for Lifelong Health and Ageing33 Bedford Place London WC1B 5JU UK

## Abstract

**Background:**

Few studies of the association between psychiatric disorder and premature death have adjusted for key confounders and used structured psychiatric interviews. We aimed to investigate if psychiatric disorder was associated with a higher risk of mortality and whether any excess mortality was due to suicide, or explained by other health or socioeconomic risk factors.

**Methods:**

We used data from the MRC National Survey of Health and Development, a nationally representative UK birth cohort. 3283 men and women completed the Present State Examination at age 36. The main outcome measure was all-cause mortality before age 60.

**Results:**

Those with psychiatric disorder at age 36 had a higher risk of death even after adjusting for potential confounders (Hazard ratio = 1.84, 95% C.I. 1.22-2.78). Censoring violent deaths and suicides led to similar results.

**Conclusions:**

Psychiatric disorder was associated with excess premature mortality not explained by suicide or other health or socioeconomic risk factors.

## Background

Many studies have shown an association between psychiatric disorder and premature death[1-5], mainly from cardiovascular disease[6-9] and suicide[9-11]. However negative findings [12,13] have been reported and questions regarding the degree to which any association may be accounted for by confounding or mediating factors remain unresolved[8]. Existing studies have significant limitations. Few are population-based[8]; follow-up periods are often short[14,15]; many have relied on subjective measures of psychiatric disorder[16-18]; and almost none have controlled for key potential mediating factors, including physical health status at baseline, tobacco and alcohol consumption[8]. We therefore have only limited information on patho-physiological mechanisms by which psychiatric disorder might lead to higher mortality.

Wulsin suggested the model study to investigate mortality associated with psychiatric disorder would be a prospective cohort design with a large community sample using structured psychiatric interviews, controlling for physical health, smoking and alcohol consumption[8]. We present findings from a study meeting all these criteria with the advantages of low attrition and follow-up over 25 years.

## Methods

The Medical Research Council National Survey of Health and Development (NSHD) is a socially stratified cohort of 5362 individuals followed up since birth in England, Scotland and Wales in March 1946. The sampling procedure and follow-up have been described in detail elsewhere[19]. 3322 (62.0%) were interviewed at age 36. Of the remaining 2040, 323 (6.0%) had died, 649 (12.1%) had emigrated, 510 (9.5%) had previously refused to participate and 558 (10.4%) were untraced.

At age 36 research nurses administered the Present State Examination (PSE) to 3293 participants at home[20]. The PSE assesses the frequency and severity of psychiatric symptoms in the preceding month and can be coded as an index of definition (PSE-ID) which ranges from 1-7. We distinguish three groups: those with an ID of 3 or more (22.0%) who typically have at least 5 psychiatric symptoms and were likely to have a psychiatric disorder; those with an ID of 2 (30.7%), who were mildly symptomatic with 1-4 symptoms; and those with a PSE-ID of 1 who had no symptoms (47.2%)[21]. Using the CATEGO computer-based classification system, we determined the presence of anxiety and depression disorders across PSE-ID categories. The highest PSE-ID category (PSE-ID= 3 or more) contained all cases of co-morbid depression and anxiety (n = 37), all cases of depression alone (n = 172), and the majority of cases of anxiety alone (n = 362). The remaining third of anxiety cases (without co-morbid depression) (n = 148) were present in PSE-ID category 2. No cases of anxiety or depression were present in PSE-ID category 1.

3283 of the 3293 men and women with a PSE-ID score were identified on the National Health Service Central Register and we were notified of all deaths. The underlying cause of death was coded according to ICD-9 or ICD-10. Deaths from "externalizing" disorders including violent accidental and suicidal deaths (ICD9 codes 291 - 292, 295 - 305, 307 - 309, 311 - 316, 570 - 571.3, 800 - 994, 1800 - 1869, 1880 - 1999 and ICD10 codes F61 - F69, K70 - K71, S00 - X99, Y85 - Y98) were identified.

Factors previously found to be associated in this cohort with premature adult mortality[22-24] were chosen as potential confounders. They included social class of origin, based on father's occupation at 4 years, and own social class at age 26 years, according to the Registrar General's 1971 classification, dichotomized to manual and non-manual. Childhood cognitive ability was assessed at age 15 using the Heim AH4 test,[25] the Watts Vernon reading test,[26] and a mathematics test; scores were standardised, summed, standardised again then categorised into fourths. Highest educational qualifications at age 26 were categorised into three groups: no qualifications; Ordinary levels (usually taken at 16); and Advanced levels (usually taken at 18 for university entry) or above.

Potential mediating variables were smoking behaviour, alcohol consumption and physical health status. Current smokers, ex-smokers and lifelong non-smokers were distinguished from information collected at adult follow-ups to age 36 years. Alcohol consumption was recorded at 36 years using a five day diary; total grams per day were calculated and categorised into fifths. Physical health status at age 36 has previously been assessed in detail[27]. Cohort members were categorised into those in the best (10.3%), intermediate (62.8%) or in worst physical health (26.9%), on the basis of measured blood pressure, lung function and body weight, self reported health problems and disability, and recent hospital admissions.

We used survival curves, obtained by the Kaplan-Meier method, to compare the cumulative death rates between 36 and 60 years for those with and without psychiatric disorder. Cox's proportional hazards models were used to investigate the relationship between psychiatric disorder and adult mortality rates. The proportional hazards assumption was checked using the spthtest function in Stata. Follow-up time (in months) was from the cohort's 36^th ^birthday until the first of death, emigration, or the end of March 2006. If death had not occurred, follow-up was treated as censored. Sex adjusted hazard ratios (HRs) for psychiatric disorder at 36 years were then further adjusted, in turn, for potential confounders and mediators. A further model included all variables. In these analyses, those with missing data on any potential confounder or mediator were assigned to a separate group. Sensitivity analyses were undertaken to compare the effects of psychiatric disorder on adult mortality risk, first including and then excluding this missing category.

We identified 22 cases of schizophrenia in the sample. All analyses were repeated, censoring for cases of schizophrenia and also censoring for deaths from 'external' causes (including accidental deaths and suicide). Analyses were performed using Stata 10.0[28]. All results presented have been weighted to adjust for the social class stratification in the original sample.

## Results

**Figure 1:**
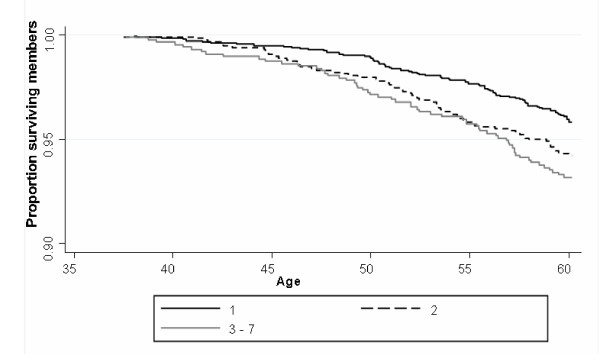
**Cumulative death rate 36-60 years by PSE-ID categories adjusted for sex**.

Between 36 and 60 years, 204 cohort members (6.2%) died. There were more deaths amongst men (7.0%) than women (5.4%) (p = 0.05). The risk of death was higher among those with psychiatric disorder (8.1%) and the less symptomatic (6.7%) compared with those with no symptoms (5.0%) (p= 0.01) (Figure 1). In univariate analyses risk of death was also higher in those whose fathers were in the manual rather than non-manual social classes or who were in the manual classes themselves in young adulthood. Lower childhood cognitive ability at 15 years and lower educational attainment at age 26 were modestly associated with mortality. Smoking and poor physical health status but not alcohol consumption was associated with premature mortality. (Results not shown: these associations are explored in this cohort in greater depth in a previous publication [24]).

Table 1 shows the hazard ratios for psychiatric disorder controlled for each of the potential explanatory factors. In all analyses there was a strong trend for those scoring higher on the PSE-ID to have a greater mortality. The hazard ratio and 95% confidence intervals (CI) for those with psychiatric disorder was 1.84 (1.22,2.78) and for the less symptomatic was 1.77 (1.20,2.61) (p = .001). In men and women, adjusting for father's social class, attained social class at age 26 years, IQ or highest educational attainment made little difference to the hazard ratios. In men, adjusting for smoking status and alcohol consumption had no effect on the hazard ratios, but adjusting for physical health status had a modest effect on the hazard ratio for the group with psychiatric disorder. In women, adjusting for smoking modestly decreased the hazard ratio in those with psychiatric disorder. After adjusting for all potential confounding and mediating variables men and women with psychiatric disorder had a higher risk death between 36-60 years, and the risk was also increased in the mildly symptomatic group. A similar pattern was found when males and females were combined - psychiatric disorder was associated with increased mortality, and the inclusion of all potential confounders and mediators only led to a modest reduction in the effect. Sensitivity analyses which excluded the missing category from each of the confounders had little effect either on the hazard ratios for psychiatric disorder or on the hazard ratios for the categories with valid values. The only exception was for the model adjusting for alcohol consumption where the number of missing cases was considerably greater than for the other confounders or mediators (see footnote on table 1). The effects of psychiatric disorder were attenuated in this restricted sample (HR (95%CI) = 1.53 (0.94,2.50) for those with psychiatric disorder and HR (95%CI) = 1.38 (0.88,2.17) for the less symptomatic, p=.063).

**Table 1:** Hazard ratios for mortality (36-60 years) by psychiatric disorder at 36 years, obtained from Cox's proportional hazards models and based on 3283 men and women and 204 deaths,

		**Hazard ratios (95%CI)**			**Test for trend P value**
		**Male**	**Female**	**All (sex adjusted)**	
**Unadjusted results**					
Psychiatric disorder - index of definition**	1	1.0	1.0	1.0	<0.001
	2	1.66 (1.02, 2.70)	1.92 (1.04, 3.57)	1.75 (1.19, 2.56)	
	3 - 7	1.84 (1.05, 3.22)	2.24 (1.22, 4.11)	2.00 (1.34, 3.00)	
Adjusted for father's social class at 4 years*					
Psychiatric disorder - index of definition**	1	1.0	1.0	1.0	<0.001
	2	1.64 (1.01, 2.67)	1.96 (1.05, 3.66)	1.74 (1.19, 2.54)	
	3 - 7	1.82 (1.04, 3.19)	2.20 (1.20, 4.05)	1.97 (1.32, 2.95)	
Adjusted for IQ at 15 years*					
Psychiatric disorder - index of definition**	1	1.0	1.0	1.0	<0.001
	2	1.67 (1.03, 2.73)	1.97 (1.06, 3.66)	1.78 (1.21, 2.60)	
	3 - 7	1.80 (1.02, 3.18)	2.26 (1.22, 4.16)	1.99 (1.33, 2.99)	
Adjusted for educational qualifications by 26 years*					
Psychiatric disorder - index of definition**	1	1.0	1.0	1.0	<0.001
	2	1.67 (1.02, 2.73)	1.98 (1.07, 3.66)	1.78 (1.22, 2.62)	
	3 - 7	1.88 (1.07, 3.29)	2.24 (1.22, 4.11)	2.04 (1.36, 3.06)	
Adjusted for social class at 26 years*					
Psychiatric disorder - index of definition**	1	1.0	1.0	1.0	<0.001
	2	1.71 (1.05, 2.79)	1.97 (1.06, 3.66)	1.80 (1.23, 2.64)	
	3 - 7	1.93 (1.10, 3.40)	2.20 (1.20, 4.04)	2.04 (1.36, 3.06)	
**Adjusted for social class, IQ, Education and adult social class at 26 years**					
Psychiatric disorder - index of definition**	1	1.0	1.0	1.0	<0.001
	2	1.70 (1.03, 2.80)	1.95 (1.05, 3.61)	1.80 (1.23, 2.65)	
	3 - 7	1.86 (1.05, 3.29)	2.11 (1.15, 3.88)	2.00 (1.33, 3.00)	
Adjusted for smoking up to 36 years*					
Psychiatric disorder - index of definition**	1	1.0	1.0	1.0	0.001
	2	1.68 (1.02, 2.76)	1.99 (1.07, 3.69)	1.75 (1.19, 2.56)	
	3-7	1.81 (1.03, 3.16)	2.01 (1.07, 3.75)	1.90 (1.26, 2.86)	
Adjusted for alcohol consumption at 36 years*					
Psychiatric disorder - index of definition**	1	1.0	1.0	1.0	<0.001
	2	1.68 (1.03, 2.74)	1.90 (1.02, 3.55)	1.75 (1.19, 2.57)	
	3 - 7	1.93 (1.11, 3.38)	2.20 (1.20, 4.04)	2.02 (1.35, 3.01)	
Adjusted for physical health status at 36 years *					
Psychiatric disorder - index of definition**	1	1.0	1.0	1.0	0.001
	2	1.59 (0.98, 2.58)	1.99 (1.08, 3.65)	1.72 (1.18, 2.51)	
	3 - 7	1.70 (0.96, 3.01)	2.19 (1.19, 4.01)	1.87 (1.25, 2.81)	
**Adjusted for all**					
Psychiatric disorder - index of definition**	1	1.0	1.0	1.0	0.001
	2	1.70 (1.02, 2.83)	2.16 (1.15, 4.02)	1.77 (1.20, 2.61)	
	3 - 7	1.80 (1.01, 3.22)	2.03 (1.09, 3.75)	1.84 (1.22, 2.78)	

* missing data for father's social class (n = 254), IQ at 15 years (n = 462), educational qualifications by 26 years (n = 149), alcohol consumption at 36 years (n = 864), social class at 26 years (n = 225), smoking up to 36 years (n = 251) and physical health status at 36 years (n-61)

** Index of definition: 1 = no symptoms; 2 = 1-4 symptoms; 3-7 = 5 or more symptoms

Table 2 shows that there was hardly any change to the overall hazard ratio when the thirty deaths from violent, accidental or suicidal causes were censored in the model. The hazard ratio for those with psychiatric disorder was slightly attenuated in men but strengthened in women. There was also little change to the overall hazard ratio when the 22 individuals with schizophrenia were excluded.

**Table 2:** Hazard ratios for mortality (36-60 years) by psychiatric disorder at 36 years, obtained from Cox's proportional hazards models and based on 3283 men and women and 204 deaths

	**Hazard ratios (fully adjusted) (95%CI)**			**Test for trend P value**
	**Male**	**Female**	**All (sex adjusted)**	
Psychiatric disorder - index of definition**				
1	1.0	1.0	1.0	0.001
2	1.70 (1.02, 2.83)	2.16 (1.15, 4.02)	1.77 (1.20, 2.61)	
3 - 7	1.80 (1.01, 3.22)	2.03 (1.09, 3.75)	1.84 (1.22, 2.78)	
Psychiatric disorder - index of definition** (Excluding schizophrenia cases*)				
1	1.0	1.0	1.0	0.002
2	1.74 (1.04, 2.91)	2.20 (1.17, 4.13)	1.81 (1.22, 2.67)	
3 - 7	1.78 (0.99, 3.20)	1.98 (1.06, 3.69)	1.81 (1.19, 2.75)	
Psychiatric disorder - index of definition** (censoring external causes)				
1	1.0	1.0	1.0	0.002
2	1.75 (1.01, 3.03)	2.62 (1.37, 5.02)	1.91 (1.27, 2.90)	
3 - 7	1.67 (0.88, 3.16)	2.26 (1.17, 4.36)	1.84 (1.18, 2.86)	
Psychiatric disorder - index of definition** (censoring external causes and excluding schizophrenia cases *)				
1	1.0	1.0	1.0	0.003
2	1.80 (1.04, 3.12)	2.69 (1.39, 5.18)	1.96 (1.29, 2.98)	
3 - 7	1.64 (0.86, 3.13)	2.20 (1.13, 4.31)	1.80 (1.15, 2.82)	

*22 cases of schizophrenia (of whom 3 had died) were excluded from model 2 and 4.

** Index of definition: 1 = no symptoms; 2 = 1-4 symptoms; 3-7 = 5 or more symptoms

## Discussion

This prospective population-based study showed that men and women with psychiatric disorder at age 36 have a greater chance of dying before age 60 than those with no psychiatric disorder. Despite controlling for a wide range of potential mediators and confounders, the effect persisted: those with psychiatric disorder had a mortality risk 84% higher than those with no disorder. This effect size is in keeping with other studies [2,3]. There was evidence for a trend across severities of psychiatric disorder with milder (and very common) symptoms being associated with an intermediate mortality risk. The association was present across the whole period of follow up. It is remarkable that an interview on mood administered just once at age 36 years appears to impact on mortality more than two decades later, and indeed the effects sizes associated with psychiatric morbidity are on a par with those shown previously[24] in the same sample for smoking status. The continued impact of psychiatric disorder at age 36 on mortality over mid-life suggests that the effect is not simply mediated by unmeasured (and uncontrolled) confounding by physical disease at baseline.

This study has a number of methodological strengths. The NSHD is a representative sample of the post-war generation born in Britain in 1946 and has a comparable pattern for premature adult mortality [29]. There is little attrition. Participants have been followed up into late middle age and even at age 53 was still representative of the national population of a similar age[30]. It includes detailed measures of psychiatric disorder at age 36. Death was independently confirmed and we have complete follow-up status on all except those who emigrated.

Several limitations should be considered. Although our measures of socio-demographic status are valid there may be some misclassification - these are "snapshots" at one time and may not fully represent the individual's background. Our measures of alcohol consumption and physical health status are taken at age 36 only and cannot give a 'whole life exposure' picture. We were therefore unable to compare disease incidence rates during follow-up for those with and without psychiatric disorder which may explain the variation in mortality. Particular caution is required in interpreting the results where alcohol consumption was included as a potential mediator as in the sample with complete data the effect of psychiatric disorder on mortality risk was somewhat smaller. This may be due to selection bias in those completing 5-day diet diaries. We have only included deaths from age 36 onwards. The role of psychiatric disorders in the 322 deaths before this is not known although Lee (2006) has shown in this cohort that high trait anxiety is associated with reduced risk of accidental death before 36[31]. There were only 11 suicides amongst the cohort between the ages of 16 and 50[32]. Given that we used only a single measure of psychiatric disorder the hazard ratio we have calculated probably represents an underestimate of the true association between psychiatric disorder and mortality.

How might psychiatric disorder increase the risk of premature death? At least four hypotheses deserve consideration. First, psychiatric disorder might be indirectly associated with higher mortality via risk behaviours not analysed here - lower exercise, greater obesity or poor compliance with medication. There is increasing evidence that patients with severe mental illness receive less good physical health care than others [33,34]. Second, there may be direct biological links whereby immunological or endocrine effects associated with depression increase the risk of death from cardiovascular disease or cancer[7,35]. Third, it is possible that the effects observed relate not to the psychiatric disorders but to the effects of their treatment, for example diabetes in patients taking antipsychotic medication[36]. Fourth, the association between psychiatric disorder and mortality might represent a common 'upstream' cause such as adverse intrauterine exposures[37] or genetic pleiotropy[38,39].

## Conclusions

Our study has confirmed the increased mortality in those suffering from psychiatric disorder. Moreover it has demonstrated that this association cannot be accounted for by suicide, smoking, alcohol or worse general physical health although these are important public health issues in this vulnerable group. We believe this is the first time that this has been shown in a cohort containing data on all the major potential confounding variables.

Health inequalities are of increasing public health and policy interest. That psychiatric disorders are associated with death is insufficiently recognized by patients, healthcare professionals and policymakers. Our finding that this is an independent effect only emphasizes the importance that should be attached to good mental healthcare. We have highlighted a number of possible mechanisms to explain this association and further, more detailed analyses are required to disentangle these possible pathways and thereby develop potential interventions.

## Competing interests

All authors declare that they have no competing interests.

## contributors

MJH, MHH and DK conceived and designed the study. IS and RH undertook the data analyses. MJH drafted the manuscript, and MHH, RD and DK critically revised the manuscript for important intellectual content. All authors read and approved the final manuscript.

## Pre-publication history

The pre-publication history for this paper can be accessed here:

http://www.biomedcentral.com/1471-244X/11/37/prepub
